# A decision tree model for predicting mediastinal lymph node metastasis in non-small cell lung cancer with F-18 FDG PET/CT

**DOI:** 10.1371/journal.pone.0193403

**Published:** 2018-02-27

**Authors:** Kyoungjune Pak, Keunyoung Kim, Mi-Hyun Kim, Jung Seop Eom, Min Ki Lee, Jeong Su Cho, Yun Seong Kim, Bum Soo Kim, Seong Jang Kim, In Joo Kim

**Affiliations:** 1 Department of Nuclear Medicine of Pusan National University Hospital, Busan, Republic of Korea; 2 Biomedical Research Institute of Pusan National University Hospital, Busan, Republic of Korea; 3 Department of Internal Medicine of Pusan National University Hospital, Busan, Republic of Korea; 4 Department of Thoracic and Cardiovascular Surgery of Pusan National University Hospital, Busan, Republic of Korea; 5 Department of Internal Medicine of Pusan National University Yangsan Hospital, Yangsan, Republic of Korea; 6 Research Institute for Convergence of Biomedical Science and Technology of of Pusan National University Yangsan Hospital, Yangsan, Republic of Korea; 7 Department of Nuclear Medicine of Pusan National University Yangsan Hospital, Yangsan, Republic of Korea; Peter MacCallum Cancer Centre, AUSTRALIA

## Abstract

We aimed to develop a decision tree model to improve diagnostic performance of positron emission tomography/computed tomography (PET/CT) to detect metastatic lymph nodes (LN) in non-small cell lung cancer (NSCLC). 115 patients with NSCLC were included in this study. The training dataset included 66 patients. A decision tree model was developed with 9 variables, and validated with 49 patients: short and long diameters of LNs, ratio of short and long diameters, maximum standardized uptake value (SUVmax) of LN, mean hounsfield unit, ratio of LN SUVmax and ascending aorta SUVmax (LN/AA), and ratio of LN SUVmax and superior vena cava SUVmax. A total of 301 LNs of 115 patients were evaluated in this study. Nodular calcification was applied as the initial imaging parameter, and LN SUVmax (≥3.95) was assessed as the second. LN/AA (≥2.92) was required to high LN SUVmax. Sensitivity was 50% for training dataset, and 40% for validation dataset. However, specificity was 99.28% for training dataset, and 96.23% for validation dataset. In conclusion, we have developed a new decision tree model for interpreting mediastinal LNs. All LNs with nodular calcification were benign, and LNs with high LN SUVmax and high LN/AA were metastatic Further studies are needed to incorporate subjective parameters and pathologic evaluations into a decision tree model to improve the test performance of PET/CT.

## Introduction

According to Korean cancer statistics, lung cancer is the leading cause of cancer death and expected to account for 22.6% of all cancer deaths[1]. The 5-year survival rates was improved dramatically from 11.3 to 21.9% for 2 decades[1]. However, 15% are diagnosed as localized stage with confined to the primary site, 22% as regional with spreading to regional lymph nodes (LN), and 57% as distant with cancer metastasized[2]. To determine the optimal therapies among surgery, chemotherapy and radiotherapy, TNM staging is one of the most reliable tools that stratify the disease[3].

For pretreatment evaluation, imaging modalities of positron emission tomography/computed tomography (PET/CT) and CT are recommended for non-small cell lung cancer (NSCLC) regardless of stage[3]. As there is the fundamental limits of spatial resolution in PET, CT may have superiority in T staging[4]. However, in lung cancer with localized or regional disease, the status of mediastinal or hilar LNs is the most significant factor to determine TNM stage. Dwamena et al. meta-analyzed the diagnostic performance of both CT and PET in N staging, showing the accuracy of 92% by PET[5]. Recently, study by Pak et al. has updated the diagnostic performance of PET/CT in NSCLC with the pooled sensitivity of 62% and the pooled specificity of 92%[6]. In addition, studies from tuberculosis endemic countries showed lower sensitivity and specificity[6]. Thus, in a certain clinical setting, nuclear medicine physicians often encounter the ambiguous cases related to tuberculosis and inflammatory diseases in evaluating mediastinal LNs. Therefore, we aimed to develop a decision tree model to improve diagnostic performance of PET/CT to detect metastatic LNs in NSCLC.

## Materials and methods

### Patients

A total of 115 patients with NSCLC (adenocarcinoma or squamous cell carcinoma) were included in this study. F-18 FDG PET/CT was done before lobectomy, or pneumonectomy with LN dissection between July 2009 and December 2013. The training dataset included 66 patients from Pusan National University Yangsan Hospital in Yangsan, Korea. The validation dataset (n = 49) was collected from Pusan National University Hospital in Busan, Korea. Clinical data of age, sex, pathologic report of each lymph node and lung cancer, American Joint committee on Cancer staging, and location of lung cancer were recorded. All data were fully anonymized before analyzing. This retrospective study was reviewed and approved by the Institutional Review Board of our institution (PNUH-2016-0032).

### F-18 FDG PET/CT

According to the standard protocol of our hospital, patients were injected intravenously 5.18 MBq/kg of F-18 FDG after fasting for at least 6 h with blood glucose level <140 mg/kg. PET/CT scans were started 60 min after injection. Image acquisition was done with the same protocol and the same PET/CT scanners (Biograph 40, Siemens, Knoxville, TN, USA) in both hospitals. During image acquisition, a CT scan was obtained first for attenuation correction, and an emission scan was consecutively obtained from the skull base to the proximal thigh. PET images were reconstructed using an iterative algorithm (ordered-subset expectation maximization) with image matrix size of 256×256.

### Image analysis

F-18 FDG PET/CT datasets were reviewed by 2 experienced nuclear medicine physicians with Syngo software provided by Siemens. Only LNs with pathologic reports were included in measuring diameters (short and long), the maximum standardized uptake value (LN SUVmax), and mean Hounsfield unit (HU). To compare FDG uptake of LNs with the blood pool activity, a region of interest was drawn within the wall of the ascending thoracic aorta (AA) and superior vena cava (SVC) approximately every 4mm on 5 consecutive axial images[7]. Subsequently, LN SUVmax was divided by average of 5 SUVmax of each AA (LN/AA) and SVC (LN/SVC). In addition, the ratio of diameters dividing long axis with short was calculated (L/S) to characterize the shape of LNs.

### Statistical analysis

Pathologic report from surgery was considered as a gold-standard in this study. Chi-square test, Fisher’s exact test and Mann-Whitney test were used to determine statistical differences between training set and validation set. A decision tree model was developed by R version 3.1.1 (The R Foundation for Statistical Computing). Multiple line graphs of frequency of metastasis were drawn by MedCalc v.9.3 software (MedCalc, Mariakerke, Belgium).

## Results

### Characteristics of patients and LNs

A total of 301 LNs of 115 patients were evaluated in this study. In training set, 66 patients with 172 LNs were included, while 49 patients with 111 LNs in validation set. Patient characteristics are demonstrated in Table 1.

**Table 1:** Characteristics of patients.

	Training dataset	Validation dataset	p
Patients	66	49	
Age (range, years)	66 (34–79)	65 (44–74)	0.2710
Sex, n (%)			0.7506
- Male	41 (62.1)	29 (59.2)	
- Female	25 (37.9)	20 (40.8)	
Pathology, n (%)			0.5407
- ADC	45 (68.2)	36 (73.5)	
- SCC	21 (31.8)	13 (26.5)	
Location, n (%)			0.4884
- RUL/RML/RLL	16 (24.2) /10 (15.2) /11 (16.7)	19 (38.8)/5 (10.2)/7 (14.3)	
- LUL/LLL	18 (27.3)/11 (16.7)	13 (46.9)/5 (10.2)	
TNM staging, n (%)			0.0042
- 1	30 (45.5)	37 (62.7)	
- 2	14 (21.2)	8 (16.3)	
- 3	17 (25.8)	4 (8.2)	
- 4	5 (7.6)	0 (0)	

ADC, adenocarcinoma; SCC, squamous cell carcinoma; RUL, right upper lobe; RML, right middle lobe; RLL, right lower lobe; LUL, left upper lobe; LLL, left lower lobe.

None of LNs with nodular calcification was proved to be metastatic in both dataset. After excluding LNs with nodular calcification (n = 18), LNs of both dataset were compared in Table 2.

**Table 2:** Characteristics of lymph nodes after excluding nodular calcification (n = 18).

	Training dataset (n = 172)			Validation dataset (n = 111)		
	Benign	Metastatic	p	Benign	Metastatic	p
LNs (n)	138	34		106	5	
Diameter (mm)						
- Short	6.1 (2.6–13.3)	8.2 (3.4–30.5)	0.0002	5.6 (2.4–14.4)	8.2 (5.0–15.3)	0.0709
- Long	9.9 (3.2–26.0)	13.4 (1.0–37.0)	0.0260	10.5 (3.7–23.6)	10.0 (9.2–17.5)	0.5269
- Ratio (Long/Short)	1.6 (1.0–4.3)	1.5 (1.1–3.9)	0.1640	1.8 (1.0–4.0)	1.4 (1.1–2.0)	0.1212
LN SUVmax	2.0 (1.1–7.9)	4.3 (1.2–16.5)	<0.0001	2.0 (1.0–12.4)	5.3 (2.2–15.5)	0.0046
SUV Ratios						
- LN/AA	1.3 (0.4–4.1)	2.9 (0.8–12.3)	<0.0001	1.1 (0.6–7.3)	2.8 (1.2–8.2)	0.0047
- LN/SVC	1.4 (0.6–4.7)	3.3 (0.8–12.9)	<0.0001	1.3 (0.7–8.3)	3.9 (1.6–11.3)	0.0047
Mean HU	36 (2–83)	36 (1–80)	0.7307	33 (1–81)	48 (20–52)	0.4510

LN, lymph node; SUV, standardized uptake value; AA, ascending thoracic aorta; SVC, superior vena cava; HU, hounsfield unit

Thirty-four LNs of training set (19.7%) and 5 of validation set (4.5%) were proved to be metastatic after surgery. Metastatic LNs had a larger short diameter for training dataset (p = 0.0002) and the trend in validation dataset (p = 0.0709). Higher LN SUVmax, and SUV ratios (LN/AA and LN/SVC) were measured in metastatic LNs for both training and validation datasets. Mean HU was not significantly different between benign and metastatic LNs in both datasets.

### Decision tree model

**Fig 1:**
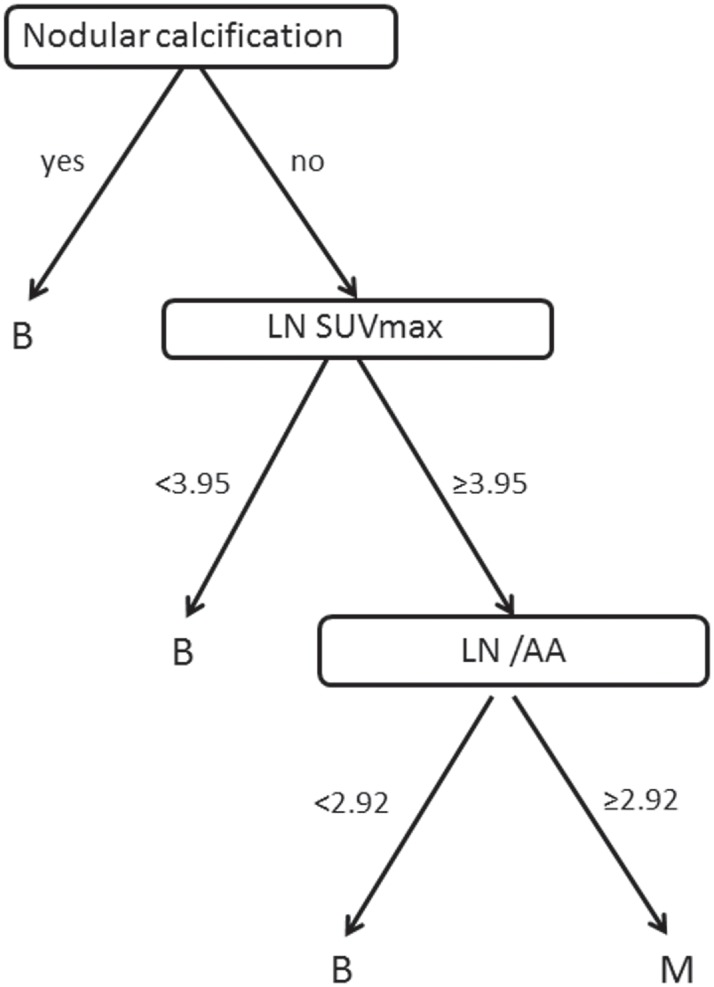
A decision tree model. The model consists of 3 decision nodes: nodular calcification, LN SUVmax, and LN/AA. Nodular calcification was applied as the initial imaging parameter, and LN SUVmax was assessed as the second. LN/AA was required only to high LN SUVmax (≥3.95).

A decision tree model was developed with training dataset. A total of 9 variables were included: short and long diameters of LNs, L/S, LN SUVmax, mean HU, LN/AA, and LN/SVC. The model consists of 3 decision nodes: 1) nodular calcification, 2) LN SUVmax, and 3) LN/AA (Fig 1).

**Fig 2:**
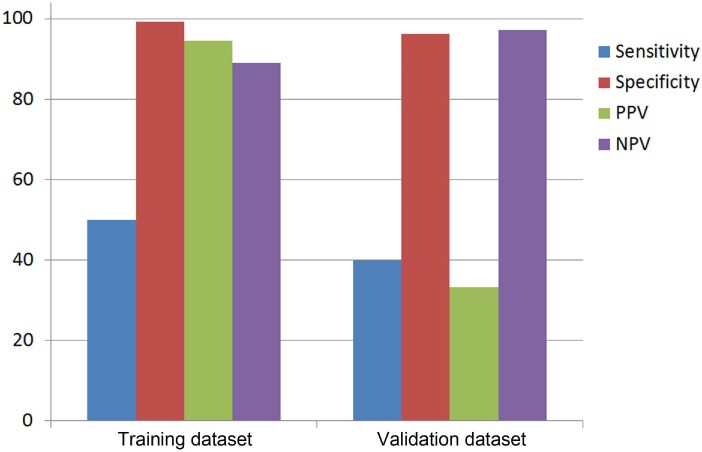
Diagnostic performance of a decision tree model with both training and validation datasets.

Nodular calcification was applied as the initial imaging parameter, and LN SUVmax was assessed as the second. LN/AA was required only to high LN SUVmax (≥3.95). The diagnostic performance of a decision tree model for each dataset is presented in Fig 2. Sensitivity was 50% for training dataset, and 40% for validation dataset. However, specificity was 99.28% for training dataset, and 96.23% for validation dataset.

## Discussion

According to the decision tree model, specificity was above 95%, while sensitivity was 50% or lower. Although several parameters were evaluated to be included in a decision tree model, 2 PET parameters (LN SUVmax and LN/AA) and nodular calcification were selected as main decision nodes.

TNM staging at presentation in NSCLC has the great impact on both treatment selection and prognosis. In this study, we included the data from the integrated PET/CT without contrast enhancement. To check the best performance of integrated PET/CT in nodal staging solely, we did not include data from CT chest with contrast enhancement in each LN. We measured and extracted several parameters of mediastinal and hilar LNs, which were pathologically confirmed by surgery after PET/CT. Both PET parameters (LN SUVmax, SUVmax of ascending aorta, and SUVmax of superior vena cava) and CT parameters (short diameter, long diameter, nodular calcification, and mean HU) were measured and ratios were calculated, which were incorporated into a decision tree model. Among them, nodular calcification, LN SUVmax, and a ratio of LN SUVmax and SUVmax of ascending aorta were included in a decision model. LN SUVmax, so called classical parameter of PET, showed a discriminating power with a cutoff value of 3.95 in this study. In cases of high LN SUVmax (≥3.95), LN/AA with cutoff value of 2.92 was adopted to discriminate metastasis from benign LNs. Previous studies have reported several cutoff values of LN SUVmax. Arbitrary cutoff values of 2.5[8, 9] or 3.5[10] and those from receiver-operating characteristics[11] were adopted to detect metastatic LNs. In a decision tree model, LN with LN SUVmax lower than 3.95 was considered as benign, which might be a higher cutoff value than classically adopted. However, neither short nor long diameters were included in a decision tree model, different from our expectations. To characterize benign LNs, calcification and high attenuation (>70HU) were used in previous studies[10, 12, 13]. In this study, all LNs with nodular calcification were benign LNs. However, mean HU was not significantly different between benign and metastatic LNs in both training and validation dataset. Therefore, interpreting LNs with mean HU>70 as benign might produce several false negatives.

There might be several reasons to explain this low sensitivity with this decision tree model. First, we included LNs with pathologically confirmation after surgery. Firstly, micrometastatic disease was detected at surgery, however not in PET/CT due to its limitation of resolution, which tends to detect disease no smaller than 6-8mm. Secondly, although nuclear medicine physicians and radiologists interpret scans with both objective and subjective parameters such as visual assessment of LNs, which cannot be measured quantitatively, we included only the objective parameters from PET/CT in this study. Therefore, the sensitivity of PET/CT might be disappointing for nodal staging without the help of subjective parameters. In addition, false positives in mediastinal LNs on PET/CT are well known such as tuberculosis lymphadenitis, granulomatous lymphadenitis[14]. However, studies on false negatives in mediastinal LNs on PET/CT has not been evaluated thoroughly. Micrometastasis from primary lung cancer or partial volume effect might be related with these false negatives[15, 16]. False negatives on PET/CT may lead to underestimate N staging in NSCLC. Nuclear medicine physicians may interpret bilateral symmetric FDG uptake of mediastinal and hilar LNs as chronic inflammatory process, and miss metastatic LNs hidden among the concurrent infection or inflammation[6]. Further studies to improve sensitivity of PET/CT in the setting of bilateral symmetric FDG uptake in chronic inflammatory process should be evaluated. There are several limitations in this study. First, although we enrolled patients from 2 institutions, a small number of patients could be included. In addition, although we enrolled patients sequentially from both hospitals, there was a difference in staging of lung cancer. This can be a weakness of these datasets in this study. Secondly, high specificity might be achieved from high threshold of SUVmax. Third, we included patients with lung cancer of SCC and ADC in this study. Further studies will be needed including pathologies other than SCC and ADC.

In this study, although sensitivity is disappointing, specificity and negative predictive value are satisfactory. According to the guidelines from European Society of Medical Oncology, absence of suspicious mediastinal LN metastasis on both PET and CT scans can proceed to surgery without endoscopic techniques such as endobronchial (EBUS) or esophageal ultrasound (EUS)-guided sampling[17]. However, national comprehensive cancer network recommends that pathologic mediastinal LN evaluation (mediastinoscopy, mediastinotomy, EBUS, or EUS) are necessary before surgery regardless of findings from PET/CT[3]. There are controversies on guidelines with pretreatment evaluation on negative mediastinal LNs on PET/CT. Further studies are needed to incorporate both imaging modalities and pathologic evaluations into the protocols with best performance.

## Conclusion

We have developed a new decision tree model for interpreting mediastinal LNs on PET/CT scans. All LNs with nodular calcification was benign, and LNs with high LN SUVmax (≥3.95) and high LN/AA (≥2.92) were metastatic. Further studies are needed to incorporate subjective parameters and pathologic evaluations into a decision tree model to improve the test performance of PET/CT.

## Supporting information

S1 File Data of this study. (XLSX)
